# Atypical Hemolytic Uremic Syndrome Caused by a Rare Complement Factor B Mutation

**DOI:** 10.7759/cureus.23207

**Published:** 2022-03-16

**Authors:** Sai Samyuktha Bandaru

**Affiliations:** 1 Internal Medicine, Baton Rouge General Medical Center, Baton Rouge, USA

**Keywords:** complement factor b, hemolytic uremic syndrome, atypical hus, thrombotic microangiopathy (tma), complement-mediated

## Abstract

Thrombotic microangiopathy (TMA), a rare and diagnostically challenging condition, commonly presents with a triad of thrombocytopenia, hemolytic anemia, and end-organ damage, such as renal failure. Most cases of the hemolytic uremic syndrome (HUS) are mediated by Shiga toxin-producing *Escherichia coli*, but some cases present as an atypical HUS, which includes thrombotic thrombocytopenic purpura and complement-mediated thrombotic microangiopathy (C-TMA). Although C-TMA occurs because of genetic and acquired mutations in the complement regulatory factors, it is usually hereditary. The currently available treatment options include therapeutic plasma exchange and administration of eculizumab, which is a monoclonal antibody against C5. Here, we report a diagnostically challenging and extremely rare case of a middle-aged Caucasian man who was diagnosed with atypical HUS that was caused by a mutation in complement factor B. This case highlights the importance of not overlooking rare causes of TMAs because the diagnostic evaluation is important for guiding appropriate management and obtaining a favorable prognosis.

## Introduction

Thrombotic microangiopathies (TMAs) are usually rare and have multiple etiologies. In this report, we present a diagnostically challenging case of complement-mediated thrombotic microangiopathy (C-TMA), which is also known as an atypical hemolytic uremic syndrome (HUS). Most cases of C-TMAs are hereditary and can be caused by underlying genetic mutations that lead to the dysregulation of the complement pathway [1]. C-TMAs are caused by genetic and acquired mutations in the complement regulatory factors [2]. With timely diagnosis, they can be appropriately treated. The currently available treatment includes therapeutic plasma exchange and monoclonal antibody against C5, eculizumab [3].

## Case presentation

A 54-year-old Caucasian man was admitted to our hospital because of a rib fracture from an accident. During the hospital course, he developed sepsis from pneumonia and was found to have elevated creatinine that required hemodialysis. He had a medical history that included coronary artery disease, aortic stenosis, and hypertension. No other significant surgical or family history was noted. On examination, the patient was tachycardic, had tenderness to palpation on the right lower chest, decreased breath sounds on the right lung auscultation, and skin pallor. The rest of the physical examination was unremarkable.

**Figure 1:**
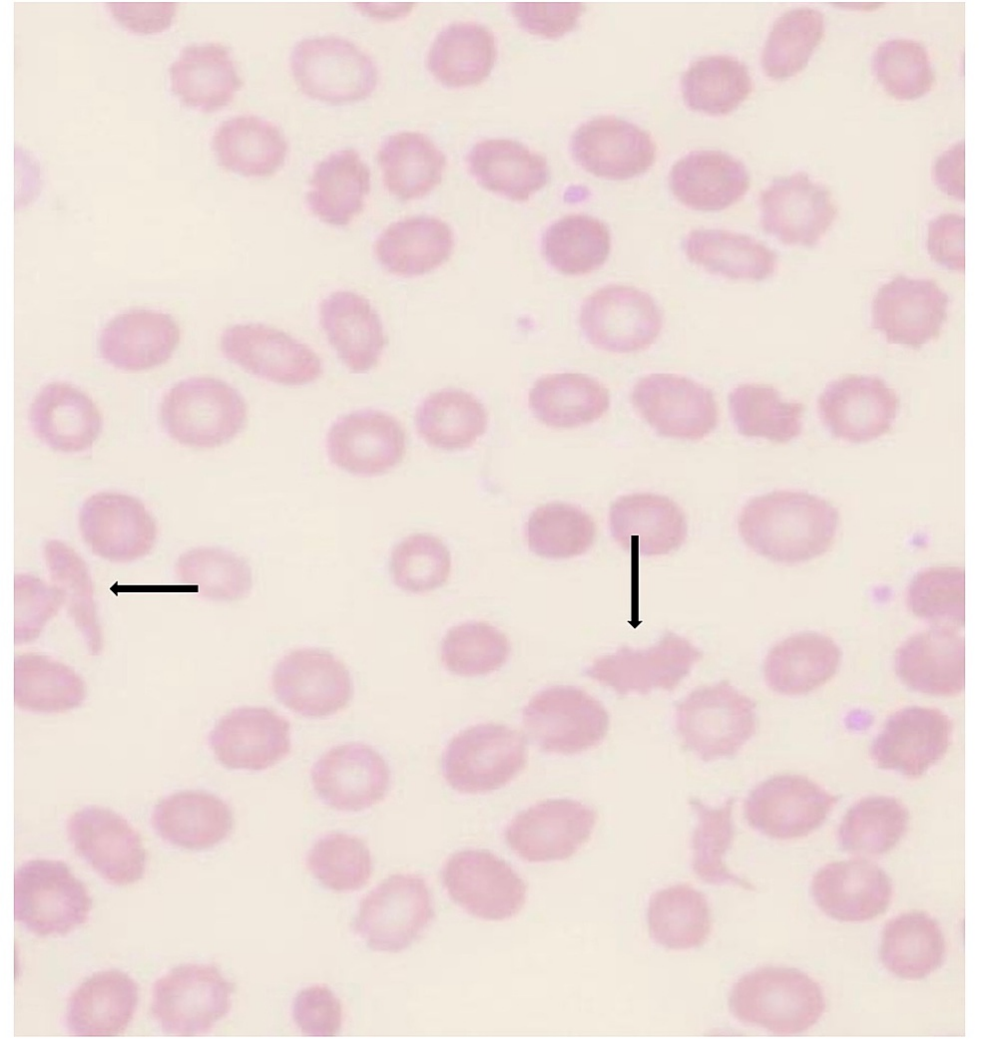
Peripheral smear showing schistocytes concerning for microangiopathic hemolytic anemia.

Further workup showed anemia with a hemoglobin level of 6.8 g/dL, haptoglobin of <8 g/dL, and lactate dehydrogenase of >700 IU/L. Coombs test was negative, and the peripheral smear showed schistocytes, which were suggestive of nonhemolytic anemia consistent with microangiopathic hemolytic anemia (Figure 1). Furthermore, he was thrombocytopenic with platelet counts ranging from 70,000 to 120,000/mL and had elevated creatinine levels ranging from 3 to 6.1 mg/dL. Eventually, his acute renal failure necessitated dependence on intermittent dialysis. 

Considering the triad of features that suggested atypical HUS, further workup was done to determine the etiology of TTM. Blood cultures, Shiga toxin, methylmalonic acid levels, B12, antineutrophil cytoplasmic antibodies, and light chain assays were negative. The ADAMTS13 level was within normal limits. Renal biopsy revealed proliferative glomerulonephritis with C3 deposits only and no immunoglobulin (Ig)G. Moreover, there was diffuse endocapillary hypercellularity with thrombi. Complement levels showed low C3 at 86 mg/dL and normal C4 at 19 g/dL. His immunofixation was positive only for C3 2± and negative for IgA, IgG, and IgM. The other workups done to rule out a post-infectious etiology of kidney failure were all negative for antistreptolysin O, anti-nuclear antibody, and anti-glomerular basement membrane. These findings suggested disruption in the regulation of the complement pathway and supported a compatible etiology for atypical HUS.

Because of high suspicion for C-TMA, genetic studies were done and showed heterozygous missense variant mutation in complement factor B (CFB), which increased the formation of C3 convertase. This confirmed the diagnosis of C-TMA. After hematology consultation, he was scheduled to receive definitive therapy with eculizumab as an outpatient.

## Discussion

In this report, a rare case of C-TMA was described. TMAs usually present with a triad of microangiopathic hemolytic anemia, renal failure, and thrombocytopenia. In some cases, this triad of features can be absent upon initial presentation. TMAs can be categorized as primary or secondary. The primary etiologies include Shiga toxin-induced TMA, which is usually known as HUS; drug-induced thrombotic thrombocytopenic purpura (TTP); and C-TMA, which is considered atypical HUS. Secondary causes are pregnancy-associated HELLP (hemolysis, elevated liver enzymes, low platelet count) syndrome; autoimmune diseases, such as systemic lupus erythematosus; infections; and malignancy [1,2].

The usual workup for TMAs should include stool cultures to rule out *Escherichia coli* o157: H7 and *Shigella*; Shiga toxin assay for patients with diarrheal illness and abdominal pain; and measurement of ADAMTS13 level to rule out TTP [4]. Other workups to rule out secondary causes include blood cultures and ANA, ANCA, and B12 levels. Complement testing can be done when C-TMA is included as a differential diagnosis [5].

C-TMA can be secondary to genetic mutations in the complement regulatory genes or to autoantibodies against complement regulatory proteins. The disease can present at any age; in adults, the more common cause is genetic than autoantibody-mediated. Genetic mutations causing atypical HUS usually have incomplete penetrance; therefore, a second hit mechanism is involved in the disease pathogenesis. Complement testing includes autoantibodies to factor H and genetic testing [6]. Genetic testing includes evaluating for *complement factor H*, *complement factor I*, *complement factor B*, *monocyte chemoattractant protein*, C3, *complement factor H related 5*, and *thrombomodulin* [7]. In this patient with diagnosed *CFB *gene mutation, the sepsis from pneumonia might have acted as the trigger or second hit through complement regulation.

In our case, the patient had a *CFB *mutation, which is extremely rare, with reported incidence ranging from 1% to 2% [8,9]. CFB is a zymogen that acts as a catalytic site for C3 convertase. Mutations in the *CFB *gene are gain of function mutations that enhance the activity of C3 convertase or increase the resistance to inactivation by complement regulators, leading to complement activation and subsequent endothelial injury [9]. This was considered to be the pathogenesis of the atypical HUS in our patient.

Anticomplement therapy with eculizumab, which is a monoclonal antibody that blocks the formation of membrane attack complex, was used for the treatment of atypical HUS [10,11]. Since its advent, cases of end-stage renal disease have been decreasing, and discontinuation of hemodialysis has been observed in some cases. Eculizumab treatment can be used in cases of confirmed C-TMA, TMA with worsening renal function where all other causes have been excluded, a high suspicion for C-TMA, and family history of C-TMA [12].

## Conclusions

The diagnosis of atypical HUS is very challenging. This case demonstrated the importance of not overlooking rare causes, such as C-TMA, because the diagnostic evaluation is extremely important for guiding the appropriate management and obtaining a favorable prognosis.
